# Supplementary material for: Role of Portion Size in the Context of a Healthy, Balanced Diet: A Case Study of European Countries
Source: Int J Environ Res Public Health. 2023 Mar 22;20(6):5230. doi: 10.3390/ijerph20065230 (PMC10049364; doi:10.3390/ijerph20065230)
Supplement: Supplementary file 1 [file ijerph-20-05230-s001.zip › ijerph-2244441-supplementary.pdf]

International Journal of
Environmental Research and Public Health

MDPI

Supplementary Materials

Role of Portion Size in the Context of a Healthy, Balanced Diet: A Case Study of European Countries

Michele O. Carruba [1], Maurizio Ragni [1], Chiara Ruocco [1], Sofia Aliverti [2], Marco Silano [3], Andrea Amico [4], Concetta M. Vaccaro [4], Franca Marangoni [2], Alessandra Valerio [5], Andrea Poli [2] and Enzo Nisoli [1,*]

[1] Center for Study and Research on Obesity, Department of Biomedical Technology and Translational Medicine, University of Milan, Via Vanvitelli, 32, 20129 Milan, Italy
[2] Nutrition Foundation of Italy, Viale Tunisia, 38, 20124 Milan, Italy
[3] Department of Food Safety, Nutrition and Veterinary Public Health, Istituto Superiore di Sanità, Viale Regina Elena, 299, 00161 Rome, Italy
[4] Health and Welfare Unit, Censis Foundation, Piazza di Novella, 2, 00199 Rome, Italy
[5] Department of Molecular and Translational Medicine, Brescia University, Viale Europa, 11, 25123 Brescia, Italy
* Correspondence: enzo.nisoli@unimi.it; Tel.: +39-02-50317116

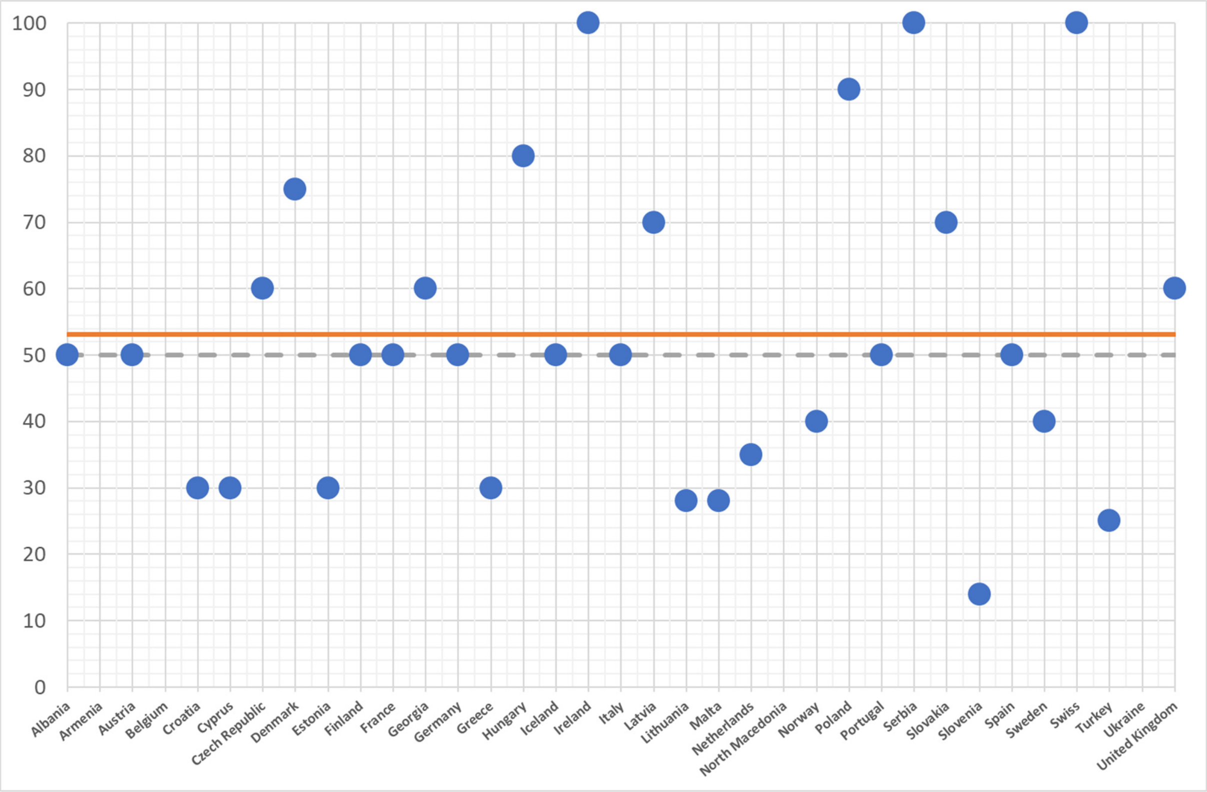

Supplementary Figure S1. Portion sizes of bread (g) in the different countries. Dashed gray line: reference standard portion defined by the Italian Society of Human Nutrition (SINU, 2014); full orange line: mean.

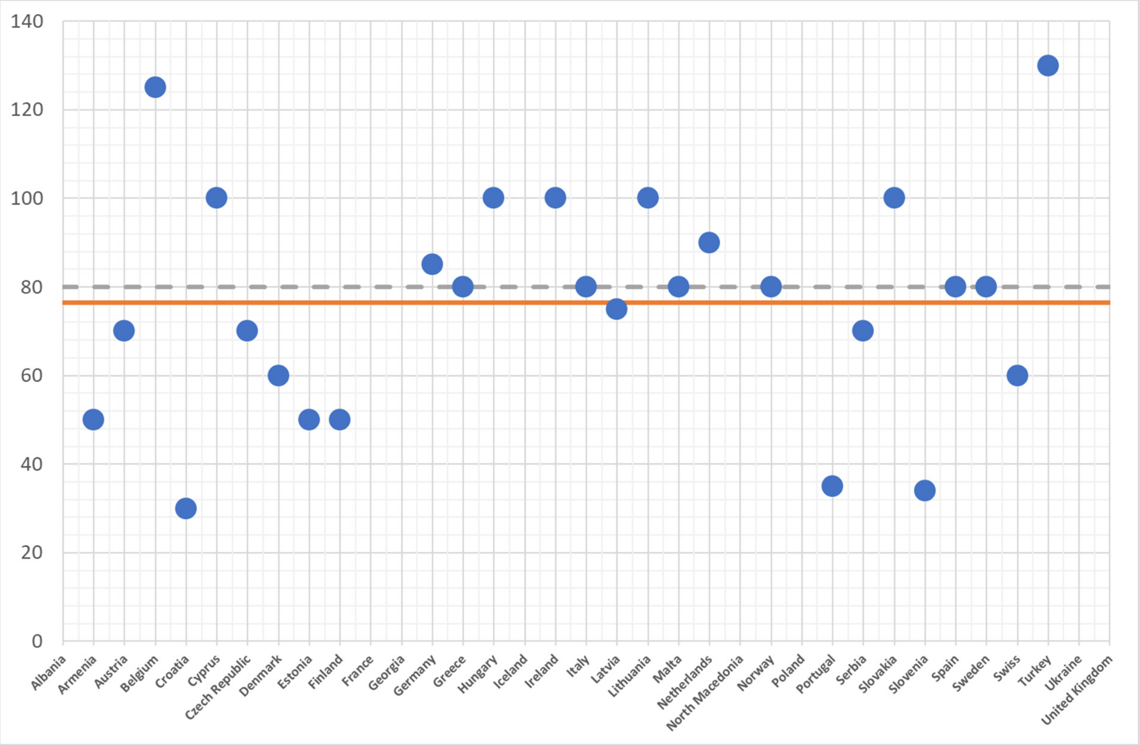

Supplementary Figure S2. Portion size of pasta & rice (raw) (g) in the different countries. Dashed gray line: reference standard portion defined by the Italian Society of Human Nutrition (SINU, 2014); full orange line: mean.

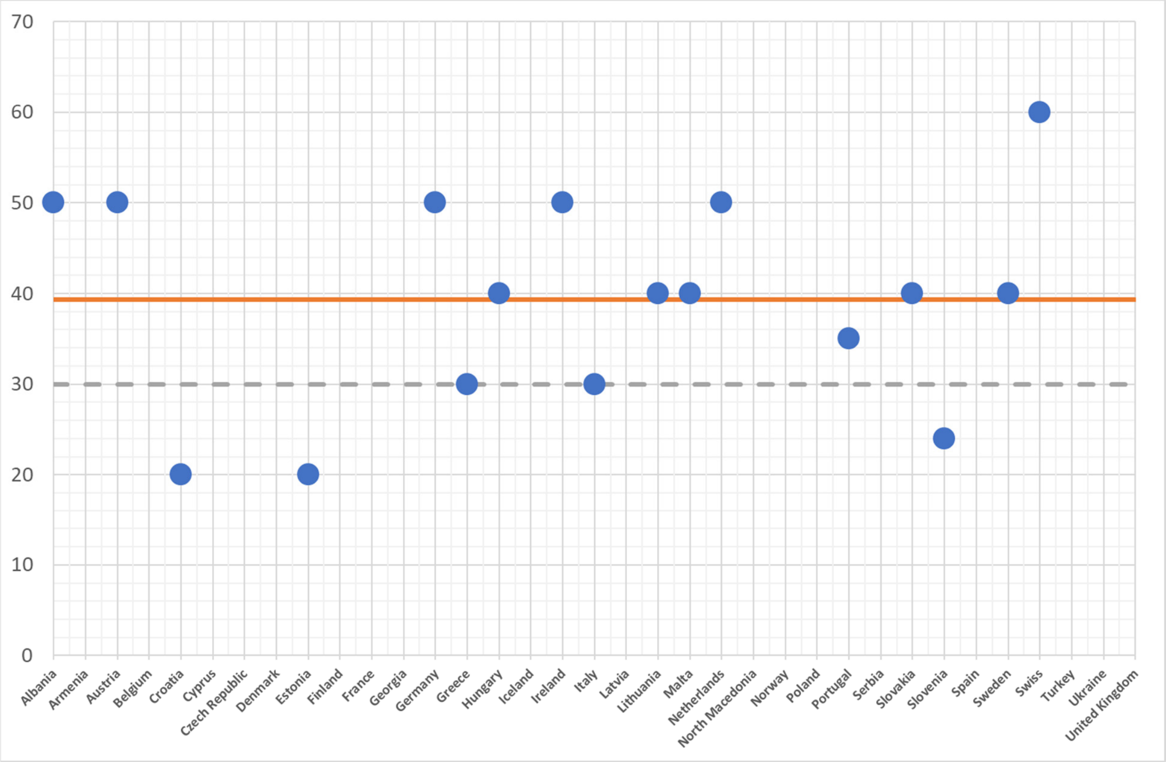

Supplementary Figure S3. Portion sizes of breakfast cereals (g) in the different countries. Dashed gray line: reference standard portion defined by the Italian Society of Human Nutrition (SINU, 2014); full orange line: mean.

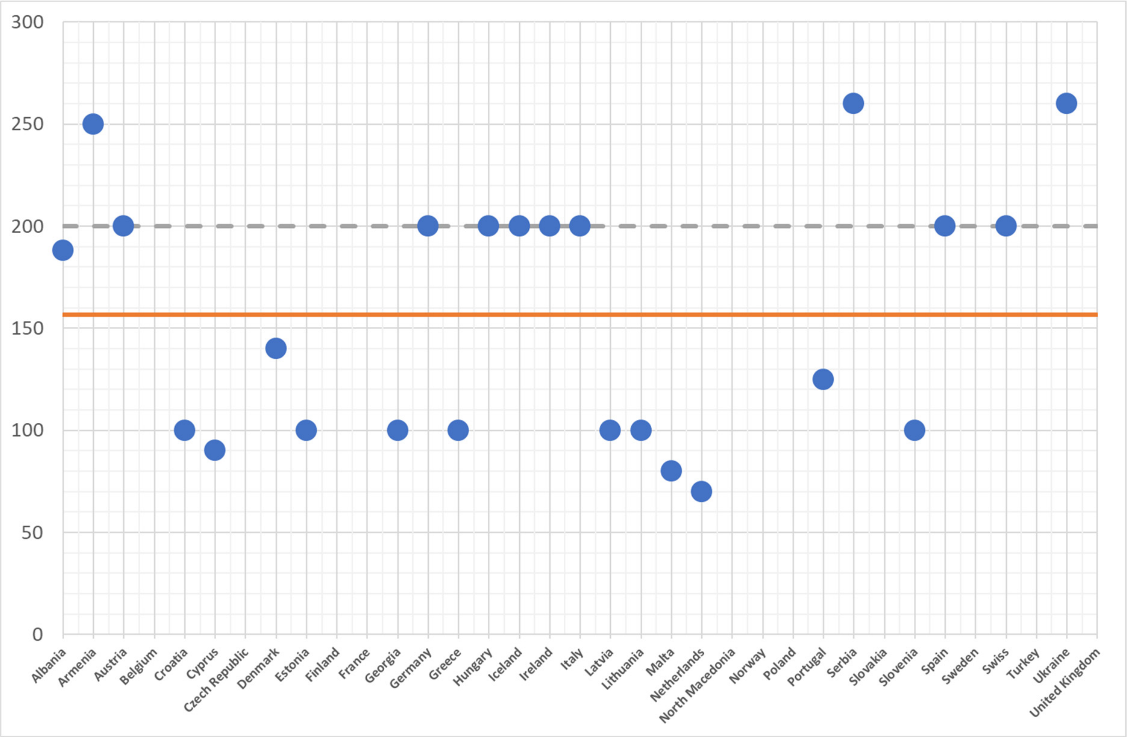

Supplementary Figure S4. Portion sizes of potatoes (g) in the different countries. Dashed gray line: reference standard portion defined by the Italian Society of Human Nutrition (SINU, 2014); full orange line: mean.

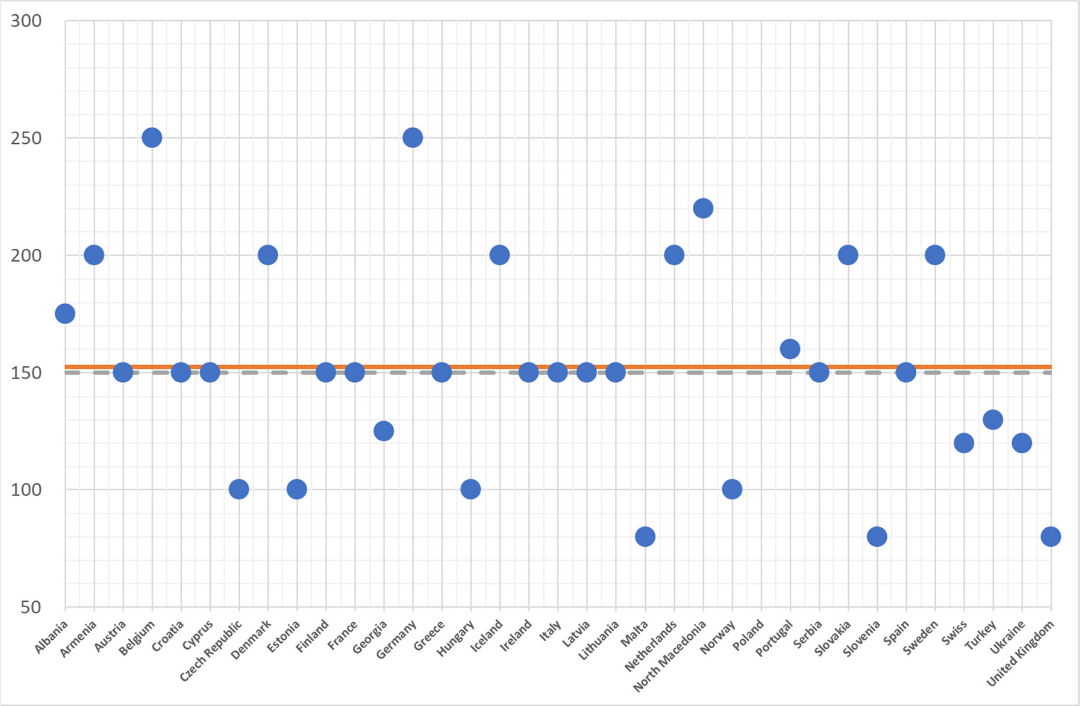

Supplementary Figure S5. Portion sizes of fresh fruit (g) in the different countries. Dashed gray line: reference standard portion defined by the Italian Society of Human Nutrition (SINU, 2014); full orange line: mean.

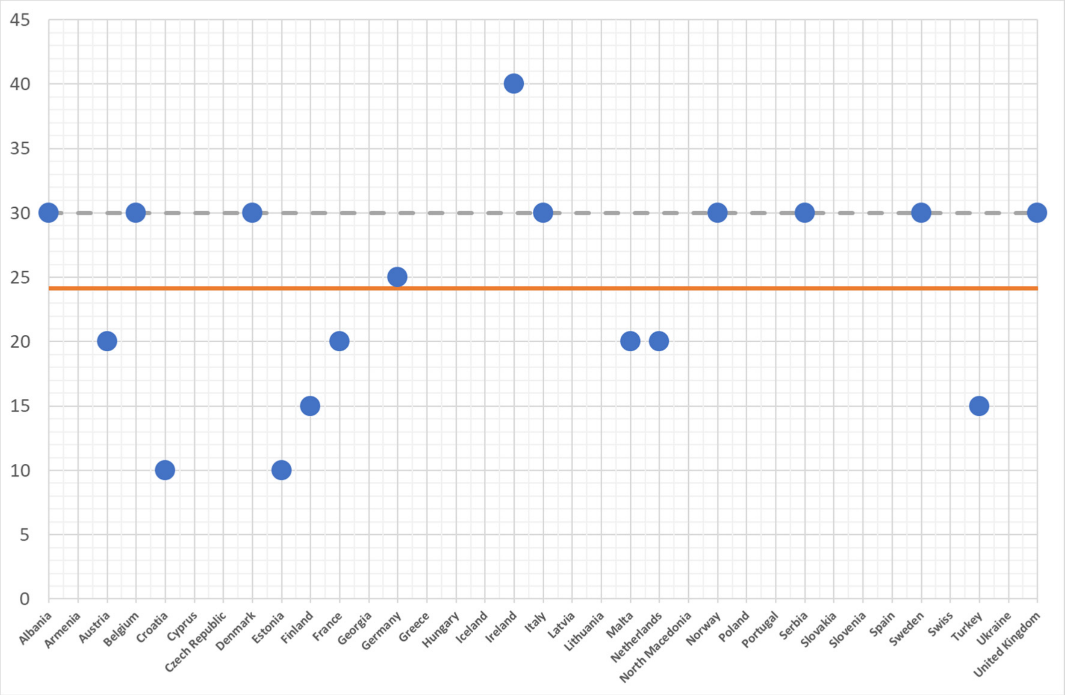

Supplementary Figure S6. Portion sizes of nuts (g) in the different countries. Dashed gray line: reference standard portion defined by the Italian Society of Human Nutrition (SINU, 2014); full orange line: mean.

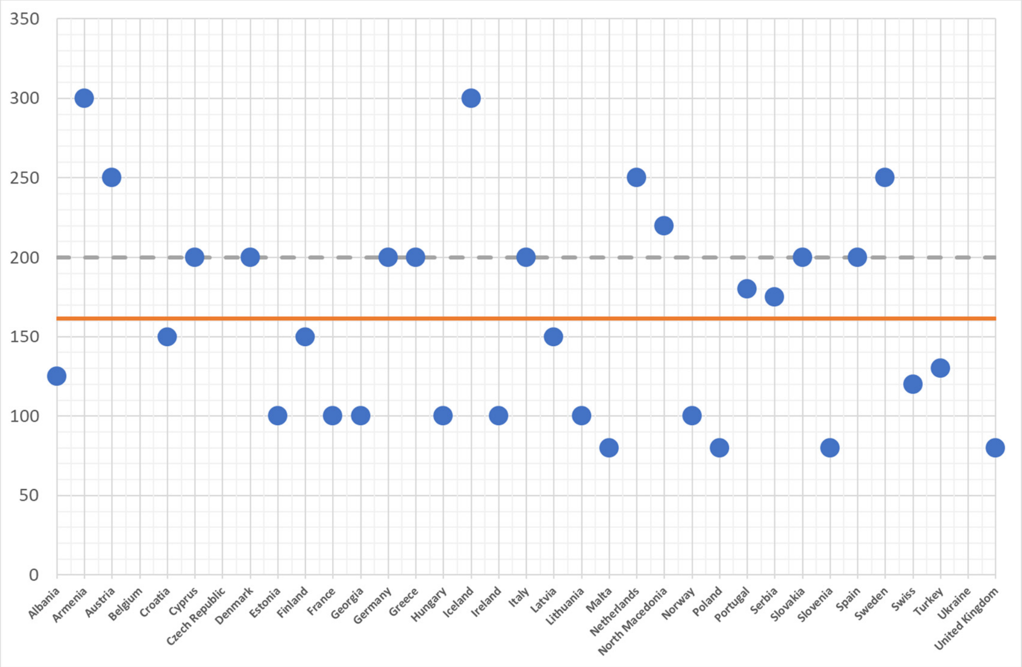

Supplementary Figure S7. Portion sizes of fresh vegetables (g) in the different countries. Dashed gray line: reference standard portion defined by the Italian Society of Human Nutrition (SINU, 2014); full orange line: mean.

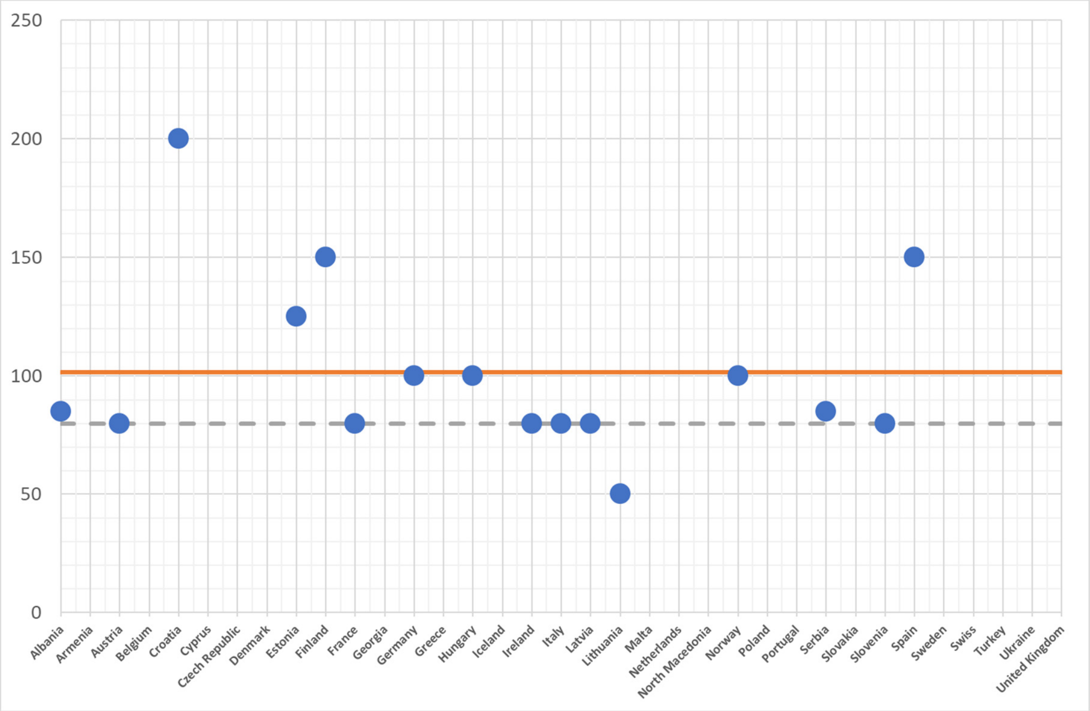

Supplementary Figure S8. Portion sizes of salad (g) in the different countries. Dashed gray line: reference standard portion defined by the Italian Society of Human Nutrition (SINU, 2014); full orange line: mean.

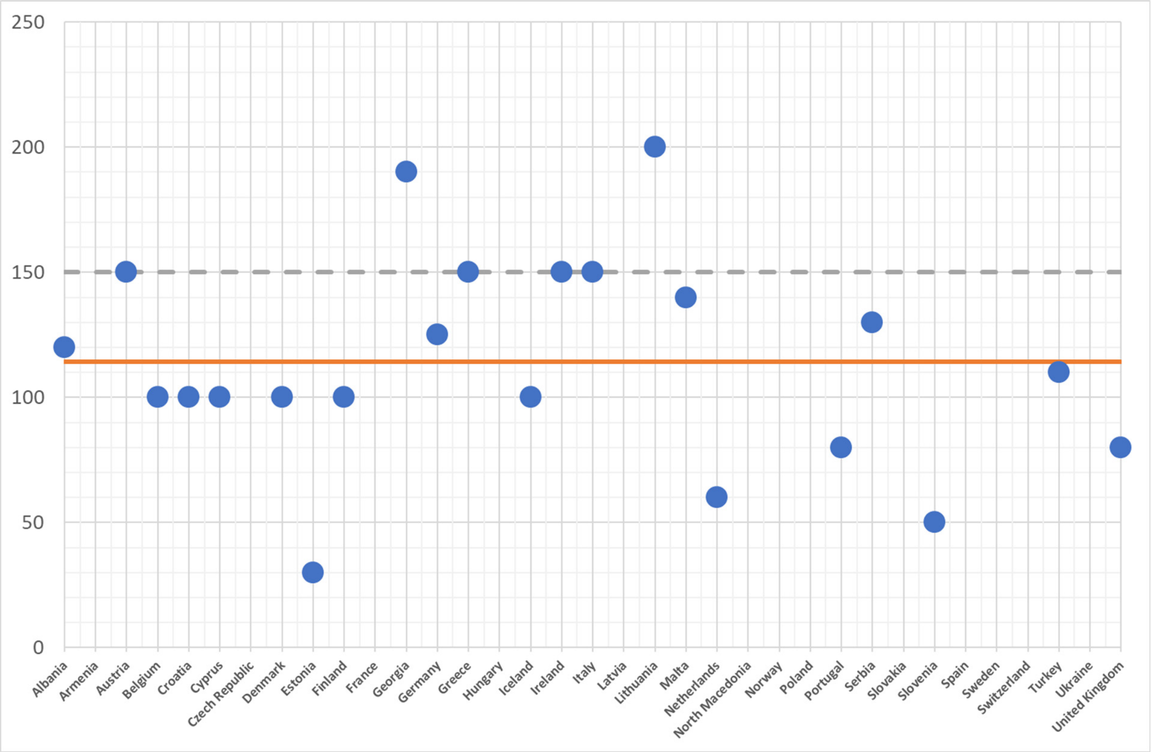

Supplementary Figure S9. Portion sizes of legumes (g) in the different countries. Dashed gray line: reference standard portion defined by the Italian Society of Human Nutrition (SINU, 2014); full orange line: mean.

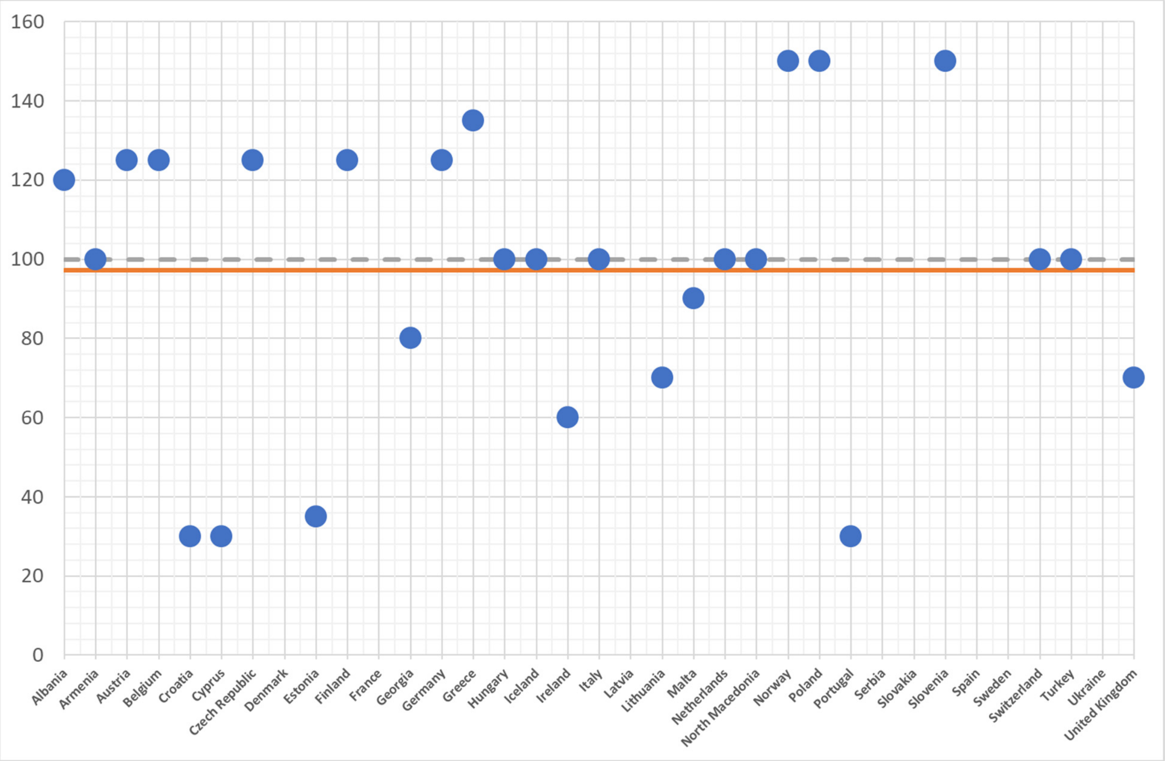

Supplementary Figure S10. Portion sizes of red meat (g) in the different countries. Dashed gray line: reference standard portion defined by the Italian Society of Human Nutrition (SINU, 2014); full orange line: mean.

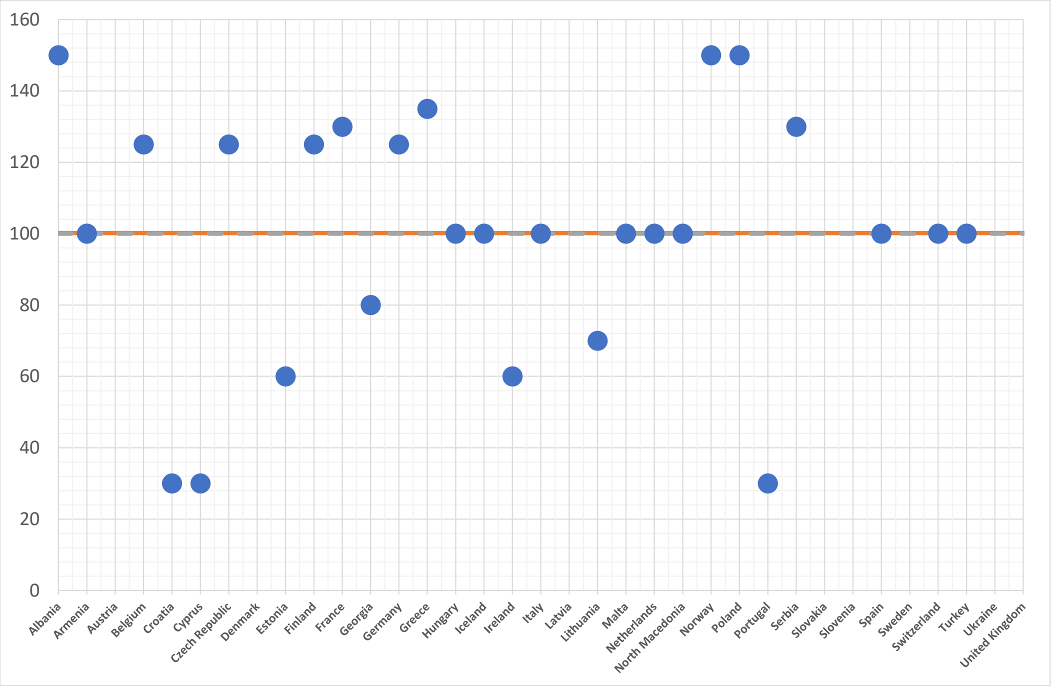

Supplementary Figure S11. Portion sizes of white meat (g) in the different countries. Dashed gray line: reference standard portion defined by the Italian Society of Human Nutrition (SINU, 2014); full orange line: mean.

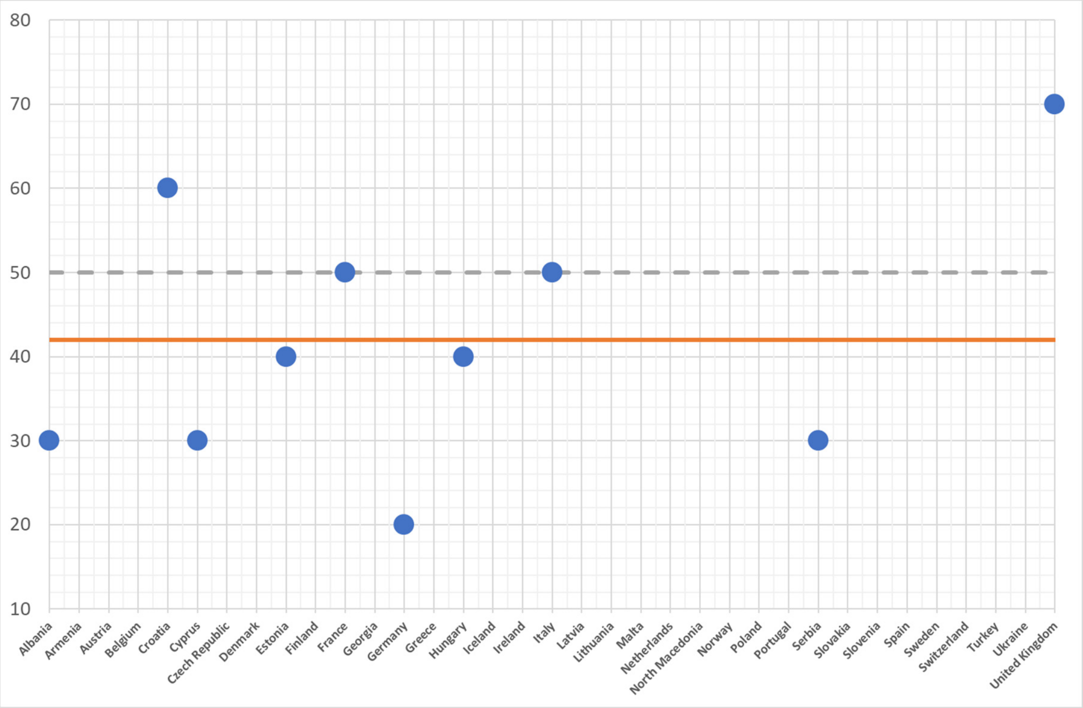

Supplementary Figure S12. Portion sizes of cured meat (g) in the different countries. Dashed gray line: reference standard portion defined by the Italian Society of Human Nutrition (SINU, 2014); full orange line: mean.

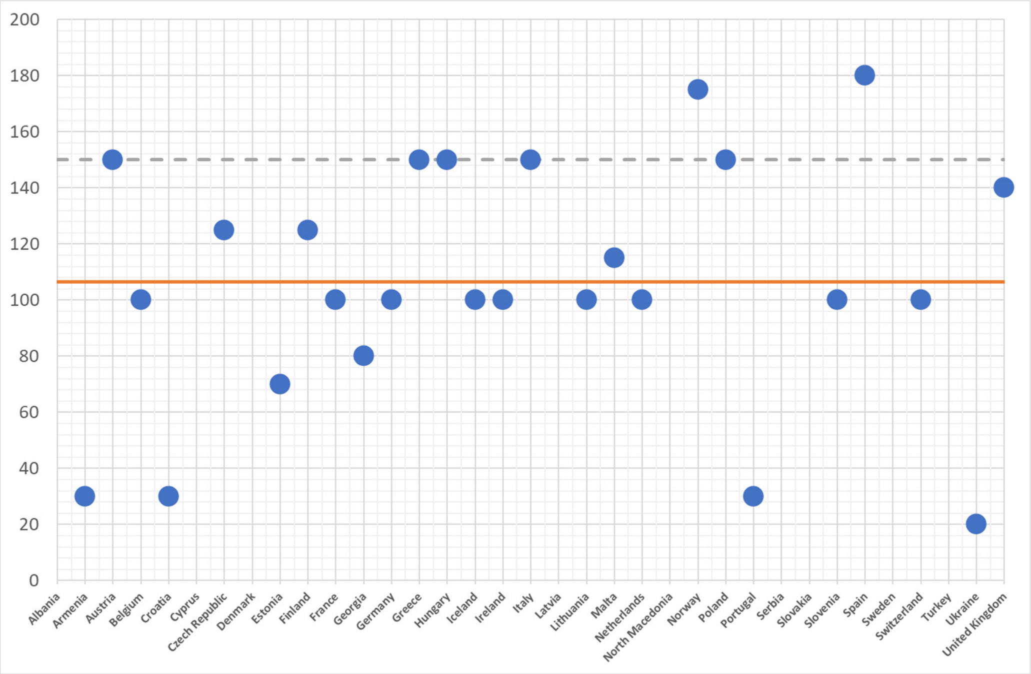

Supplementary Figure S13. Portions sizes of fish (g) in the different countries. Dashed gray line: reference standard portion defined by the Italian Society of Human Nutrition (SINU, 2014); full orange line: mean.

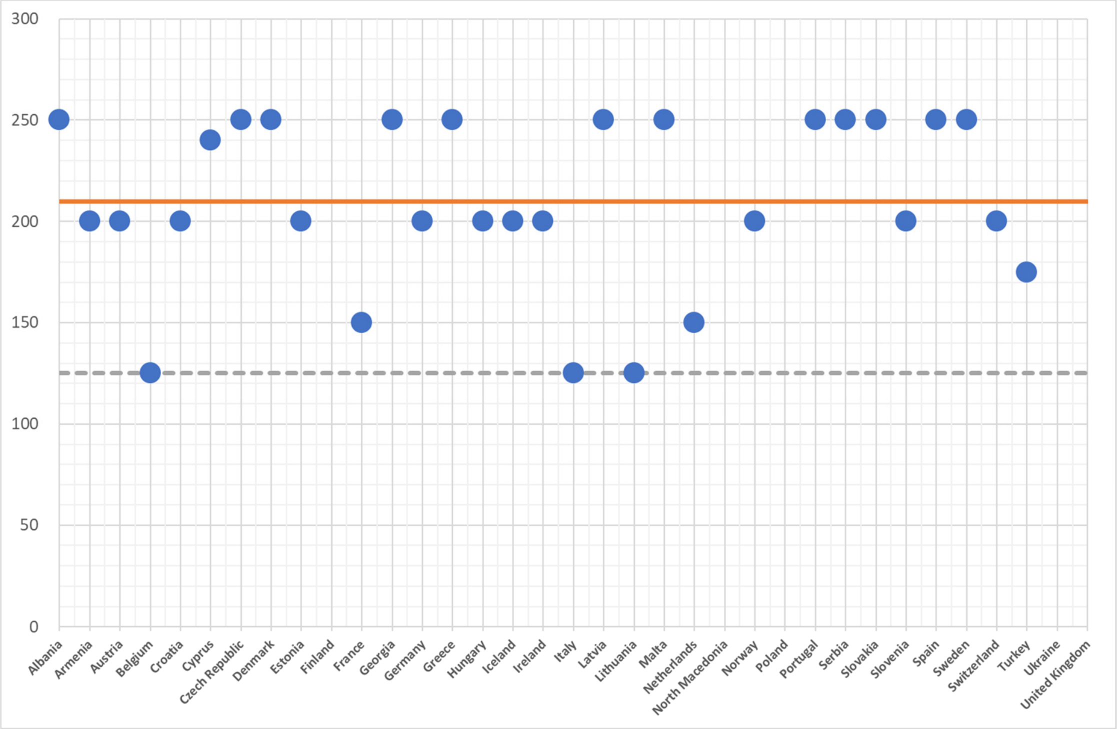

Supplementary Figure S14. Portion sizes of milk (ml) in the different countries. Dashed gray line: reference standard portion defined by the Italian Society of Human Nutrition (SINU, 2014); full orange line: mean.

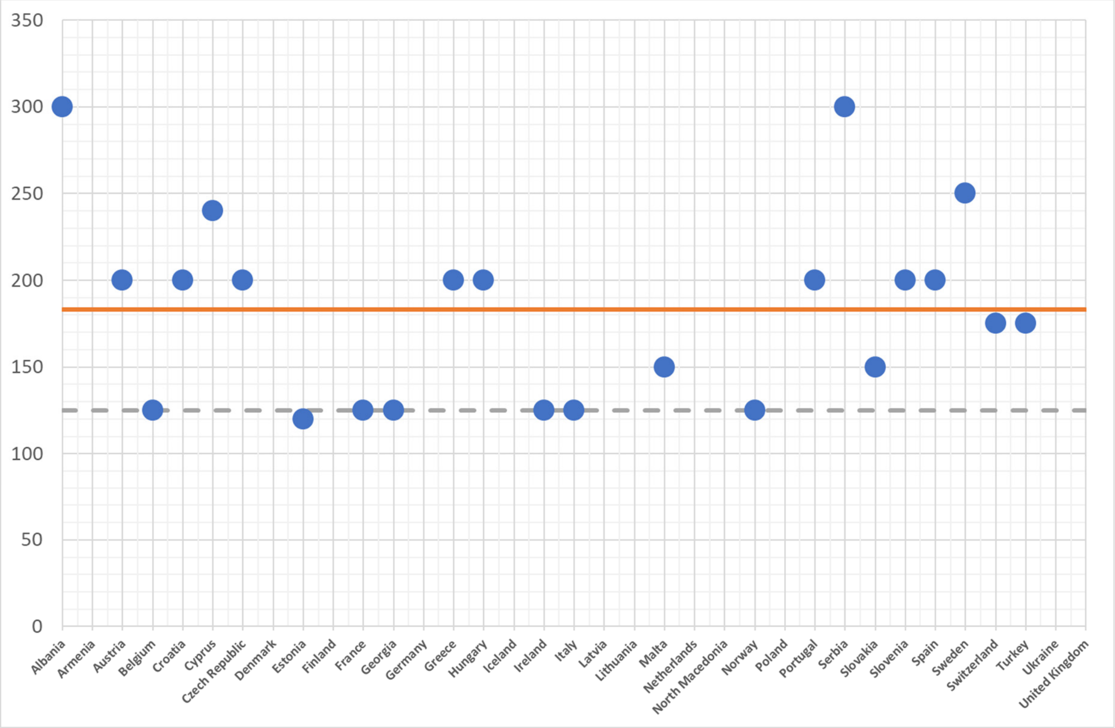

Supplementary Figure S15. Portion sizes of yogurt (g) in the different countries. Dashed gray line: reference standard portion defined by the Italian Society of Human Nutrition (SINU, 2014); full orange line: mean.

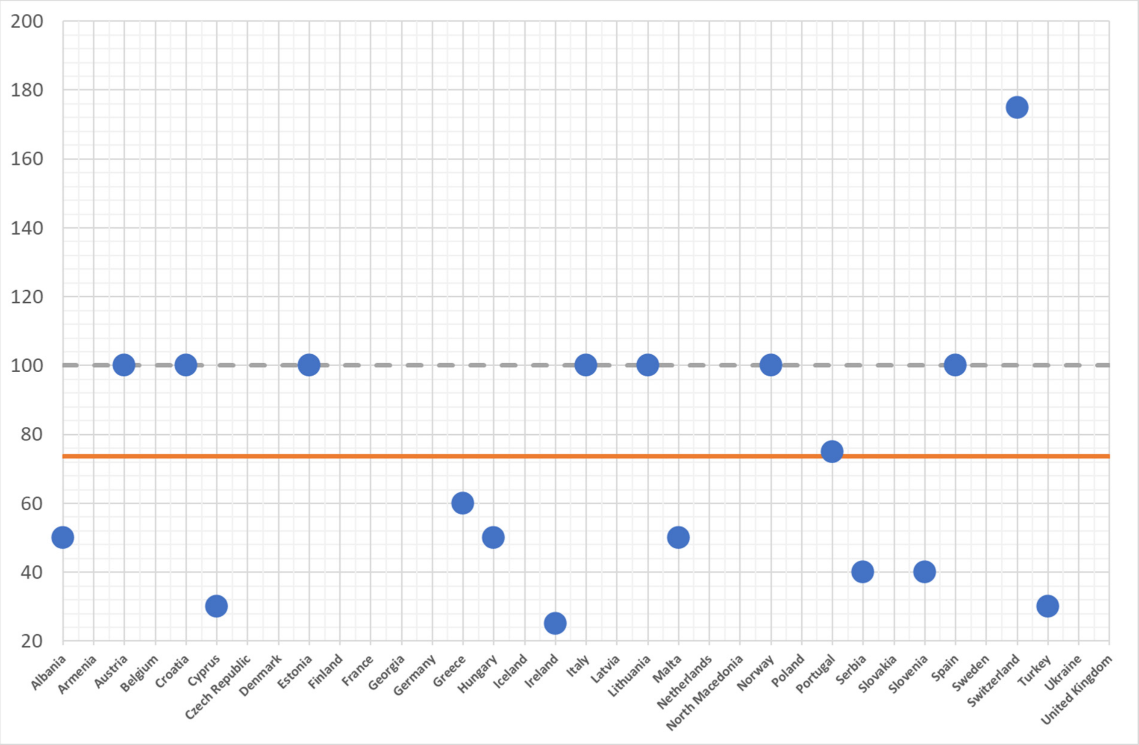

Supplementary Figure S16. Portion sizes of cream cheese (g) in the different countries. Dashed gray line: reference standard portion defined by the Italian Society of Human Nutrition (SINU, 2014); full orange line: mean.

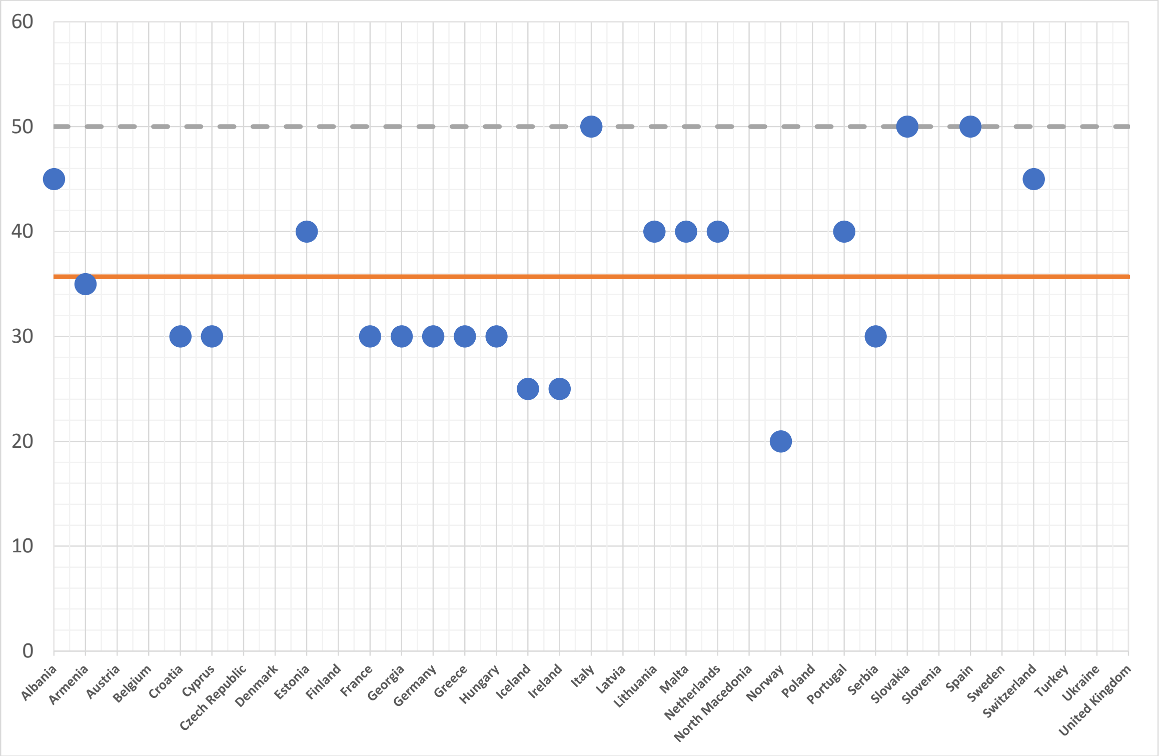

Supplementary Figure S17. Portion sizes of hard (ripened) cheese in the different countries. Dashed gray line: reference standard portion defined by the Italian Society of Human Nutrition (SINU, 2014); full orange line: mean.

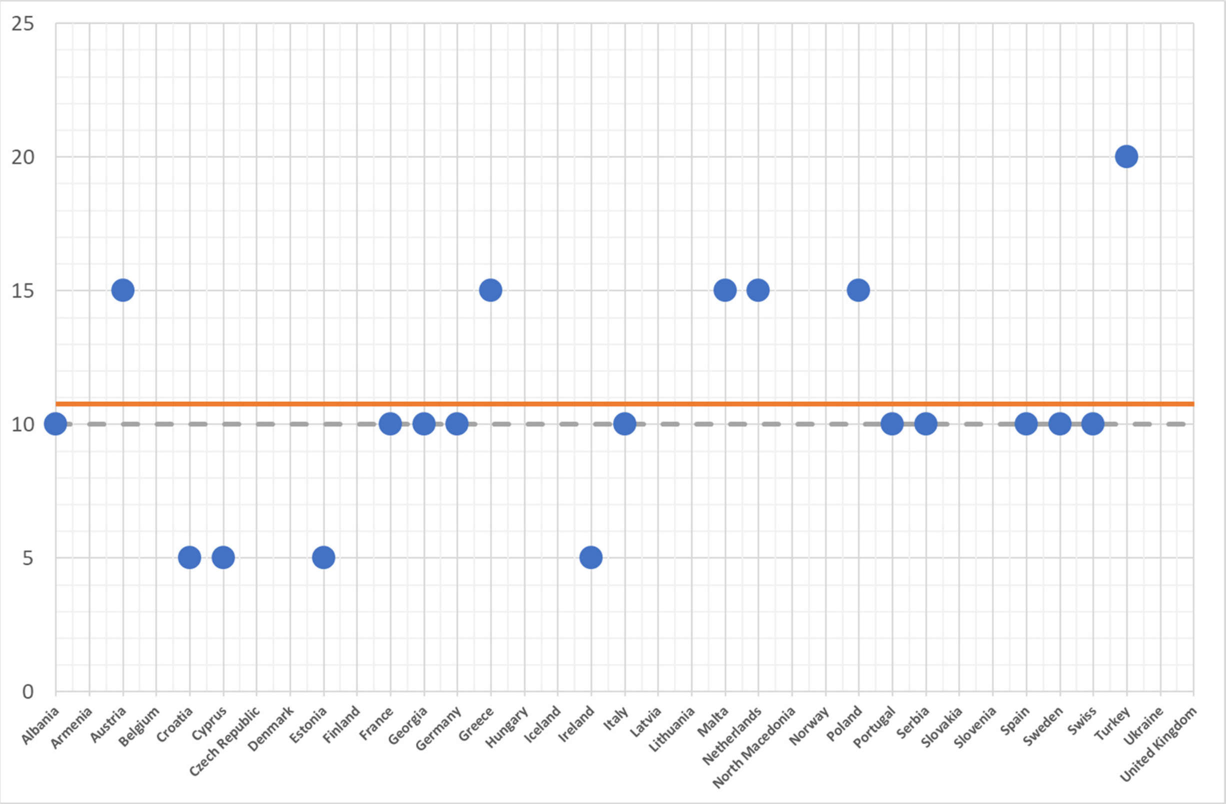

Supplementary Figure S18. Portion sizes of vegetable oils (g) in the different countries. Dashed gray line: reference standard portion defined by the Italian Society of Human Nutrition (SINU, 2014); full orange line: mean.
